# Improving Glaucoma Surgical Outcomes with Adjunct Tools

**DOI:** 10.5005/jp-journals-10028-1239

**Published:** 2018-03-01

**Authors:** Louise J Lu, Laura Hall, Ji Liu

**Affiliations:** 1 Medical Student, Department of Ophthalmology and Visual Science, Yale School of Medicine, New Haven, Connecticut, United States; 2 Ophthalmologist, Department of Ophthalmology and Visual Science, Yale School of Medicine, New Haven, Connecticut, United States; 3 Ophthalmologist, Department of Ophthalmology and Visual Science, Yale School of Medicine, New Haven, Connecticut, United States

**Keywords:** Adjunct tools, Amniotic membrane, Collagen matrix implant, Fibrin adhesive, Fibrin glue, Glaucoma, Innovations, Ologen, Surgery, Trabeculectomy.

## Abstract

Conventional glaucoma surgeries, such as trabeculectomy and glaucoma drainage device (GDD) surgery, have been enhanced by surgeons to improve outcome and decrease complications. Over the last two decades, adjuncts, such as collagen matrix implants, fibrin adhesives, and amniotic membrane transplantation (AMT) have been found to be effective in modulating fibrosis and scarring during the wound-healing process, reducing postoperative inflammation, and repairing bleb leakage or conjunctival erosion. The use of these tools provides several advantages when used in trabeculectomy, GDD surgery, and surface reconstruction associated with glaucoma surgery complications. Their use will be discussed in this review.

**How to cite this article: **Lu LJ, Hall L, Liu J. Improving Glaucoma Surgical Outcomes with Adjunct Tools. J Curr Glaucoma Pract 2018;12(1):19-28.

## INTRODUCTION

The surgical management of glaucoma has had much needed enhancements in recent years. Among these are a variety of new techniques, devices, and adjunct tools available to surgeon undertaking surgery for glaucoma. Introduced with less fanfare, but just as important, are new techniques supporting trabeculectomy and GDDs. Among the most promising of these are innovations for which there is evidence of reduced complications and improve surgical outcomes when used with trabeculec-tomy and GDD surgery.

Trabeculectomy remains the most common operation for patients with advanced glaucoma in most countries. Trabeculectomy lowers intraocular pressure (IOP) by creating a new drainage site for aqueous humor outflow underneath the conjunctiva.^1,2^ Glaucoma drainage device, or tube shunt surgery, has traditionally been reserved to treat patients with refractory cases of glaucoma who failed trabeculectomy or are at very high likelihood of failure. Results from the recent tube *vs *trabeculectomy (TVT) study indicate comparable safety and efficacy between Baerveldt 350 mm^2^ GDD and trabeculectomy in eyes with prior ocular surgery,^3,4^ though trabeculectomy still achieved superior surgical outcomes in eyes without prior ocular surgery. Trabeculectomy and GDD surgery remain the mainstay of glaucoma surgical procedures, and improvements in technique to increase predictability and decrease complications remain an active area of clinical research.

The utilization of adjunct tools for trabeculectomy and GDD surgery has the potential to improve surgical outcomes for the surgical management of glaucoma. This review will summarize the applications, techniques, and outcomes of collagen matrix implants, fibrin adhesives, and amniotic membrane transplantation (AMT) as adjunct tools for these conventional glaucoma surgeries.

## COMPLICATIONS OF CONVENTIONAL GLAUCOMA SURGERIES

The most common causes of failure of trabeculectomy are postoperative episcleral fibrosis and subconjunctival scarring. The introduction of antimetabolites, such as mitomycin C (MMC) and 5-fluorouracil (5-FU), significantly improved the success rate of trabeculectomy by acting as antifibrotic agents during wound healing and reducing scar formation after surgery.^5^ Both MMC and 5-FU act as antifibrotic agents, but MMC is used more frequently due to prolonged bleb duration and superior IOP lowering effect with MMC and the higher risk of corneal toxicity with 5-FU.^6^ However, patients experience myriad complications due to the use of MMC, including avascular filtering blebs, loss of corneal endothelial cells, thinning of the conjunctiva, bleb leaks, hypotony, and endophthalmitis.^7-11^ Early complications of trabeculectomy with MMC are often related to the surgical procedure itself, while late complications are more likely to be caused by prolonged inhibition of fibroblasts during wound healing, which promotes the formation of thin avascular blebs that are prone to leak.^12^ One retrospective study of 239 eyes estimated the probability of a bleb leak at 5 years after trabeculectomy with 0.5 mg/mL MMC to be 17.9%.^13^ A more recent retrospective study of 797 eyes after trabeculectomy performed with 0.2 to 0.4 mg/mL MMC reported that the incidence of bleb-related infection after 8 years was 3.4%.^14^ Alternatives to antimetabolites for application in trabeculectomy have recently been explored by researchers and clinicians in order to avoid the complications of MMC.

Although the TVT study suggested that GDD surgery could be an excellent alternative to trabeculectomy, there were shared and unique complications in the tube shunt surgery. Tube erosion, a potential late complication for GDDs, can lead to infection or even endophthalmitis and can be difficult to repair, especially when there is recurring tube erosion. Failure to control IOP can also be an early-to-late complication of either valved or non-valved GDDs.^15-17^ Encapsulation of the bleb around the plate of the drainage device is sometimes considered a reason for persistent IOP elevation. This could be more problematic in the valved implants, such as the Ahmed GDD. The use of antimetabolites has been studied by different groups with variable results, with some findings that MMC could increase the risk of conjunctival erosion and bleb leak.^18-21^ On the contrary, utilization of certain adjunct tools in GDD surgery showed positive results for both IOP reduction and management of complications.

## ADJUNCT TOOLS FOR CONVENTIONAL GLAUCOMA SURGERY

### Biodegradable Collagen Matrix Implant

**Figs 1A and B:**
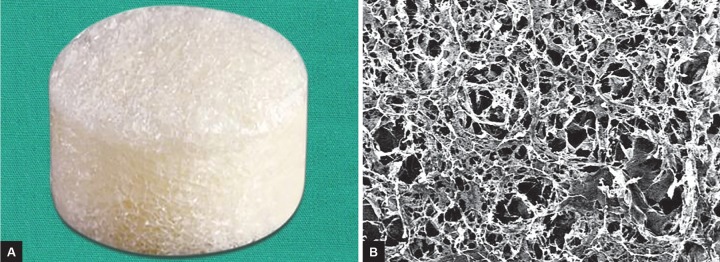
Gross appearance and electron microscopy (EM) of Ologen™ biodegradable collagen matrix implant. (A) Ologen™ disk of 6 mm in diameter × 2 mm in thickness. (B) EM photo showing type I atelocollagen biodegradable scaffold with three-dimensional porous structure. Pore diameters are between 10 and 300 μm. (Photo Credit: Aeon Astron Inc.)

One promising alternative adjuvant for trabeculectomy and GDD is Ologen™, a biodegradable three-dimensional collagen-glycosaminoglycan copolymer matrix implant that acts as a scaffold to reduce scarring during wound healing^6-8^ (Fig. 1). Olo gen“ is inserted as a spacer to separate the subconjunctival and episcleral tissues to prevent fibrosis and reorganize subconjunctival scar formation, as well as induce the growth of fibroblasts and myofibroblasts into its porous structure to secrete a loose connective tissue matrix to further promote healing.^7^ The Ologen™ implant is composed of porcine-based lyophi-lized cross-linked type I collagen and glycosaminogly-cans, with a degradation time of approximately 180 days.^7^

**Figs 2A and B:**
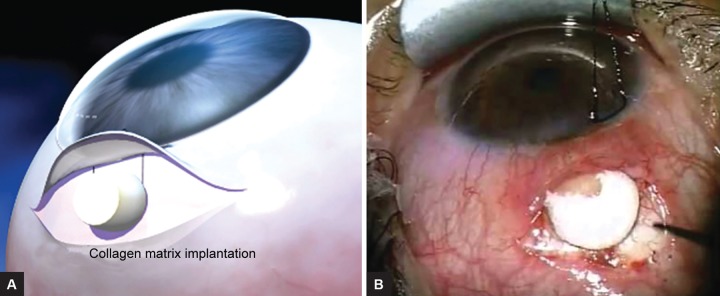
Ologen implantation for trabeculectomy: (A) Schematic diagram; and (B) surgical photo of Ologen placement showing that the implant is positioned on top of the scleral flap and under the conjunctiva to exert counterpressure on the scleral flap. (Photo Credit: Aeon Astron Inc.)

In studies with trabeculectomy, the Ologen™ implant has been positioned under the conjunctiva but on top of the scleral flap to exert counter pressure on the scleral flap, which limits transcleral filtration in the early postoperative period (Fig. 2). Although no sutures are required to close the scleral flap as the implant molds to the scleral tissue, some glaucoma surgeons recommend to use one or two scleral flap sutures when using the Ologen™ implant during trabeculectomy.^22,23^ The conjunctiva is closed to ensure complete covering of the implant and a watertight conjunctival closure.^8,24^ Similar technique was used to repair bleb leak by placing Ologen to the subconjunctival space within the filtering bleb.^22^

Early clinical studies comparing the efficacy and rates of complications using Ologen™ *vs *MMC in tra-beculectomy produced mixed results. A 2015 Cochrane Review examined eight clinical trials published up to December 2014 assessing the use of Ologen™ implant in trabeculectomy compared with MMC.^12,25-30^ The meta-analysis, which studied a total of 333 eyes of 327 participants, concluded that due to low-quality evidence, there is uncertainty whether there is a significant difference in outcomes (including IOP reduction and best-corrected visual acuity) or adverse events between the Ologen™ and MMC groups.^30^

However, more recent trials with longer patient follow-up have showed comparable or superior efficacy of Ologen™ compared with MMC; these studies were published in the past 2 years and were not included in the Cochrane Review meta-analysis. A recent retrospective study conducted by Perez et al^31^ demonstrated that trabeculectomy with Ologen had the same success rate as trabeculectomy with MMC for lowering IOP, at the 2-year follow-up mark. Perez et al^31^ analyzed IOP, bleb characteristics, and early and late postoperative complications among 65 eyes of 58 patients who underwent trab-eculectomy with Ologen by the same surgeon between 2011 and 2014. Mean IOP was reduced from 21.4 ± 9.2 to 12.3 ± 3.7 mm Hg at the last follow-up measurement (p < 0001). Notably, there were no significant late adverse effects due to Ologen at the 36-month follow-up mark and normal blebs in 86.4% of eyes at the last postoperative measurement.

The long-term effectiveness and safety of Ologen™ as an adjunct tool for trabeculectomy was evaluated by Yuan et al,^8^ who compared the Ologen™ implant with MMC among 44 patients treated with trabeculectomy. The patients were followed for 5 years and evaluated for outcomes of IOP reduction, success rate, bleb status, and frequency of adverse effects. The results indicated that Ologen™ provided higher rates of surgical success compared with MMC for glaucoma patients undergoing trabeculectomy. The mean reduction in IOP was significant in both groups at each assessment point throughout the study as well as at the final point of 5-year follow-up. Surgical success rate was significantly higher in the Ologen™ group compared with the MMC group; the complete success rate was 61% for the Ologen™ group and 31% for the MMC group (p = 0.017), and the overall success rate was 84 and 59% in the Ologen™ and MMC groups respectively (p = 0.031). Bleb height in the Ologen™ group was higher than in the MMC group in the early postoperative stage, but was similar in both groups at 5-year follow-up. Regarding bleb vascularity, the researchers reported that blebs in eyes of patients with Ologen™ implants were more vascular and diffuse (with no evidence of avascular areas) compared with eyes treated with MMC at 5-year follow-up. There was no significant difference reported in postoperative complications for trabeculectomy with either adjuvants. From these results, the study researchers concluded that the Ologen™ implant was an effective and safe alternative to MMC for improving the success rate of trabeculectomy.^8^

Results from another 5-year randomized prospective clinical trial on Ologen™ implant *vs *MMC in trabeculec-tomy were recently reported by Cillino et al,^7^ measuring IOP, bleb morphology, and frequency of complications in a cohort of 40 patients. Specifically, the primary outcome was IOP reduction, and secondary outcomes were visual acuity, mean deviation, bleb evaluation according to Moorfields Bleb Grading System (MBGS), spectral domain OCT (SD-OCT) bleb examination, number of glaucoma medications, and frequency of postoperative complications. The researchers found that mean reduction in IOP after 5 years was significant in both groups, with complete success rates for <21 mm Hg target, IOP being 65% for MMC and 70% for Ologen™. Bleb morphology and height as per MBGS score and SD-OCT analysis found no significant differences between MMC and Ologen™. Mean number of glaucoma medications was significantly reduced in both MMC and Ologen™ groups with no significant difference (p = 0.08). The MMC group reported six cystic thin avascular blebs (30%), while the Ologen™ group reported two, but no significant intergroup difference was noted (p = 0.235). The 5-year follow-up results of the study confirmed that the use of the Ologen™ implant in trabeculectomy is a safe and effective procedure for glaucoma patients, and is comparable to MMC in producing long-term success rates and efficacy in lowering IOP.^7^

El-Saied et al^23^ conducted a prospective interven-tional comparative study on the IOP lowering effect of trabeculectomy with Ologen in 40 eyes of 40 patients with refractory secondary glaucoma following failed trabecu-lectomy with MMC. Results indicated that there was a statistically significant difference (p < 0.001) between postoperative IOP compared with preoperative IOP in both patients with secondary open angle glaucoma and secondary angle closure glaucoma. In addition, postoperative blebs with Ologen were noted to be better than the blebs of the previously failed trabeculectomy with MMC (p < 0.001). The results suggest that Ologen is an effective alternative to MMC in patients undergoing repeat trabeculectomy.

Angmo et al^32^ reported a case series of 27 trabeculec-tomies with low-dose MMC (0.1 mg/mL for 1 minute) plus modified Ologen in both subscleral (1 × 1 mm) and subconjunctival (5 mm in diameter) placement for advanced glaucomatous eyes. The study found significant and persistent IOP reduction with this modified Ologen technique (38.3 ± 6.6 mm Hg preoperatively *vs *12.3 ± 1.6 mm Hg at 18 months and 12.5 ± 1.6 mm Hg at 24 months postoperatively, p < 0.0001). There was also a significant reduction in the number of ocular hypotensive medications used in this study cohort (4.2 ± 0.5 preoperatively *vs *0.07 ± 0.3 postoperatively, p < 0.0001). Additionally, the blebs showed no increased vascularity or avascular cystic appearance, which is commonly seen with higher doses of MMC application.

Larger randomized trials may confirm the efficacy of the Ologen™ implant and its advantages over MMC in reducing the risk for avascular and thin blebs. Results of future studies will continue to enhance the surgical approach for glaucoma patients to modulate wound healing and avoid the complications of antimetabolites in trabeculectomy. If the safety and tolerability of Ologen™ is upheld, perhaps the future of innovation in glaucoma surgery may also include drug-eluting biodegradable implants to further help modify wound healing and increase surgical success.^1^

Ologen™ has been shown to have the potential to be a powerful adjunct tool for glaucoma drainage implant (GDI) surgery as well. Currently, the success rate of conventional GDI surgery ranges between 60 and 90% in the 12 to 27 months after surgery with a yearly failure rate of 10% in subsequent years.^33-35^ The cumulative probability of failure after 5 years of GDD implantation ranges from 44.7 to 53% for valved Ahmed GDIs, and from 29.8 to 40% for the nonvalved Baerveldt GDDs.^4,15,16^ Surgical management after a failed GDD surgery can be improved with the use of a biodegradable collagen matrix. Studies have shown that Ologen™ can be used for repair of the conjunctiva overlying the tube of the GDD, providing a wound-healing scaffold to effectively reduce conjunctival contraction and enhance formation of the subconjunctival stroma.^33-35^

Rosentreter et al^36^ conducted an observational comparative case series of 19 patients undergoing capsule excision after failed GDD surgery; 10 of the patients were treated with capsule excision, topical MMC application, and implantation of an Ologen™ implant, while the remaining nine patients were treated by capsule excision and topical MMC alone. At the conclusion of the study, the mean IOP reduction for the capsule excision plus Ologen™ group was 12.1 mm Hg, while the mean IOP reduction for the capsule excision group alone was 8.3 mm Hg, indicating a significant difference in success rate between the two groups (p = 0.04). Additionally, none of the patients in the Ologen™ group required further pressure-reducing surgery, whereas such surgeries were deemed necessary for three of the nine patients in the non-Ologen™ group.^36^ The positive results indicate that the adjunct use of Ologen™ in capsule excision for revision of failed GDD surgery may be a safe and powerful method for improving postoperative outcomes. However, due to the relatively small sample size and observational design of the study, larger, randomized-controlled trials are required before the effectiveness of Ologen™ can be confirmed for surgical practice.

The unit cost of Ologen™ as of January 2017 is $180 according to the manufacturer’s catalog. The additional costs of utilizing an Ologen™ collagen matrix implant as an adjunct tool for glaucoma surgery can be weighed against the benefits of time saved during the procedure, safe application for certain patient populations who may have negative consequences with MMC exposure (e.g., pregnant patients), and improvement of outcomes, which remains to be further confirmed with larger clinical trials.

### Fibrin Adhesive

**Figs 3A to F:**
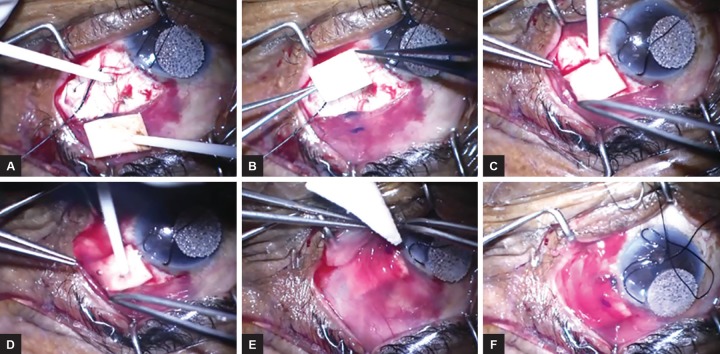
Application of the fibrin glue in the glaucoma drainage implant surgery. After the GDI was secured onto the sclera, both the surgical field and patch graft were carefully dried. Fibrinogen and thrombin were applied to the sclera and the patch graft separately (A). The patch graft was then flipped over to the scleral bed to cover the tube (B). After the patch graft was positioned and secured, fibrinogen was applied to the top of the patch graft (C), followed by thrombin (D). The conjunctiva was then approximated by using forceps. Any excessive glue was removed by a surgical sponge (E). The wound was well closed without any suture (F).

Another tool that can effectively modulate wound healing after trabeculectomy and GDIs is fibrin glue, which can be applied to the surgical surfaces for reduction of subconjunctival fibrosis and formation of a successful bleb (Fig. 3). Fibrin glue is a biological tissue adhesive that is composed of fibrinogen, factor XIII, aprotinin to inhibit fibrinolysis, and thrombin to activate factor XIII and stabilize the clot.^37,38^ It is biodegradable and has shown minimal toxicity to the ocular surface.^39-42^ The final common pathway for the intrinsic and extrinsic pathways of coagulation is mimicked by fibrin glue to achieve tissue adhesion.^37,38,43^ Currently, fibrin glue is most commonly used in ophthalmic surgery for attaching conjunctival autografts to close wounds in pterygium surgery.^44^ However, fibrin glue can also be used for conjunctival closure in strabismus surgery, vitrectomy, treatment of corneal perforations, and glaucoma surgery.^39-43,45-58^

Fibrin glue has notable application in glaucoma surgery as an adjunct tool for trabeculectomy and GDDs as well as for the repair of postoperative bleb leaks and tube erosion.^41,42^ Fibrin glue has been utilized in trab-eculectomy either by application alone or in combination with sutures to close the peritomy and scleral flap. O’Sullivan et al^41^ reported a case series of six trabecu-lectomy operations using glue alone in two eyes as well as glue plus 10-0 nylon sutures in four eyes. Notably, all eyes healed well after 2 to 7 months of follow-up and the eyes in which glue was used alone showed a nonin-flamed bleb. Another trabeculectomy study reported a case study of five eyes with fibrin glue alone to close the scleral flap and conjunctiva. No intra- or postoperative complications were observed except in one eye with a shallow anterior chamber and choroidal effusion during the early postoperative period, which resolved after medical management. All eyes achieved a functional bleb and reasonable IOP control without developing a wound leak.^48^

A larger prospective clinical trial comparing con-junctival closure with 9-0 nylon sutures (in 29 eyes) *vs *fibrin glue alone (in 28 eyes) in trabeculectomy found no differences between the two groups in IOP reduction and complication rates, except for two cases of early wound dehiscence in the fibrin glue group that required subsequent suturing.^59^ In addition, patients who received fibrin glue reported less discomfort in the first 2 weeks of the postoperative period.

The advantages of using fibrin glue in trabeculectomy include less postoperative inflammation, early postoperative flow of aqueous humor, and formation of a successful bleb. Fibrin glue effectively achieves a hemostatic effect by stopping hemorrhage and vascular leakage, as well as reduces postoperative inflammation by avoiding excessive cauterization of the sclera.^45,60^ Additionally, fibrin glue allows aqueous humor to flow freely through nonadhesive surfaces that are coated with the fibrin clot layer in the early postoperative period, as the physiological degradation of the fibrin clot into the anterior chamber takes approximately 7 to 14 days.^61^ Finally, the third advantage of fibrin glue in trabeculectomy is that it contributes to the formation of a successful bleb. Clinical studies involving procedures that utilize fibrin glue have shown that the mass of fibrin glue itself helps to decrease subconjunctival fibrosis and contribute to the formation of a successful bleb.^26,28^ Some surgeons hypothesize that coating all surfaces beneath the conjunctiva with fibrin glue may help achieve complete hemostasis to prevent bleeding or leakage from capillaries, thereby decreasing postoperative inflammation, reducing subconjunctival fibrosis, and improving free aqueous humor outflow.^43^ The main concerns of fibrin glue application without suture in trabeculectomy are the risks of postoperative wound dehiscence and outflow dynamics interruptions. More studies with long-term follow-up are needed to confirm the advantages of fibrin glue application in primary trabeculectomy.

Fibrin glue has been more widely used as an adjunct or standalone for the repair of leaking trabeculectomy blebs. The fibrin glue seals the tissues together to prevent leaks and reduces the risk of hypotony. Asrani et al^42^ performed a study in which autologous fibrin glue was used to seal a leaking bleb by applying multiple layers of glue to the carefully dried area of leakage. Nine of 12 eyes (75%) with either early or late leak were successfully repaired using this method. In another clinical study, fibrin glue was used in 10 patients who underwent bleb revision after previous trabeculectomy due to overfiltration, leak, thinning, infection, or dysesthesia. All blebs were repaired and preserved by superior con-junctival flap advancement over the bleb, which was secured with 10-0 nylon sutures and subsequent injection of fibrin glue between the conjunctival space and Tenon’s outer bleb wall. This method achieved complete success in six patients and qualified success in 1 patient.^42^ None of the patients experienced postoperative bleb leak or hypotony, and the mean IOP decreased from a preoperative mean of 13.6 mm Hg to a postoperative mean of 11.7 mm Hg.^62^

The applications of fibrin glue in GDD surgery have been assessed and found to be effective as well (Fig. 3). Kahook and Noecker^63^ conducted a retrospective case-control study of 28 patients—14 patients who underwent GDD implantation with the use of fibrin glue to secure the patch graft and conjunctiva and the remaining 14 who had the procedure with conventional sutures. There were no statistically significant differences observed in IOP reduction or rates of postoperative complications between the two groups. However, the suture group experienced a significantly higher rate of conjunctival inflammation (p = 0.002) and a greater mean time of surgery (p = 0.001) compared with the fibrin glue-assisted group.^63^ Freeman et al^58^ also assessed the effect of a similar technique using fibrin glue for GDI implantation in a pediatric population and found comparable IOP reduction and complication rates between the suture group and fibrin glue group, with significantly reduced time of surgery in the fibrin glue group. Valimaki^64^ performed a retrospective study of 34 patients who underwent GDI surgery with adjunct use of fibrin glue applied over the scleral flap when the patch graft is not used. The researchers measured mean IOP reduction among the patients to be 34.3 mm Hg preoperatively and 19.1 mm Hg at the conclusion of the study (p = 0.001) and detected no aqueous leak or postoperative complications during the follow-up time. It was hypothesized that the fibrin glue helped seal the potential leak at the sclerostomy site around the tube and prevented postoperative hypotony.

The unit cost of Tisseel fibrin sealant as of February 2017 is around $140 according to the manufacturer’s catalog. The additional costs of utilizing fibrin glue as an adjunct tool for glaucoma surgery can be weighed against the benefits of reduced time of surgery, improvement of postoperative conjunctival inflammation, and prevention of aqueous leaks; further studies are warranted to further explore the efficacy of fibrin glue as an adjunct tool in GDD surgery.

### Amniotic Membrane Transplantation

**Figs 4A and B:**
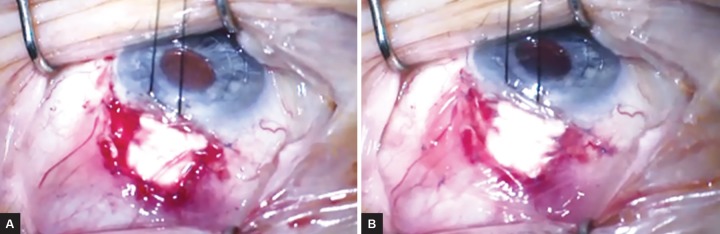
Use of double-layer amniotic membranes for tube exposure repair with inadequate conjunctival tissue. The first layer of the amniotic membrane is placed above the patch graft with the epithelial side up (A). The edge of the amniotic membrane is tucked under the conjunctiva and can be secured by either sutures or fibrin glue. Then the second layer of the amniotic membrane is placed above the first layer of the amniotic membrane and surrounding conjunctiva, and secured with interrupted sutures (B).

Amniotic membrane transplantation, with its regenerative properties, has significant potential to be utilized as an adjunct tool for ocular surgery. Human amniotic membrane has many beneficial features: it is transparent, lacks immunogenicity, and has been shown to have antiinflammatory, antifibrotic, antiangiogenic, and possibly antimicrobial properties.^65^ The tissue can be integrated into the host as a surgical graft or it can be placed temporarily as a biological bandage (Fig. 4). Amniotic membrane serves as a substrate for epithelium to grow on and suppresses tumor growth factor-p signaling to reduce fibroblast production and myofibroblast differentiation, leading to decreased scarring. In addition, it inhibits proinflammatory cytokines and traps inflammatory cells that would otherwise infiltrate the ocular surface.^65-67^

Current applications of AMT in ocular surgery are extensive, with broad applications from corneal surface disorders and perforations with and without limbal stem cell deficiency, conjunctival surface reconstruction, acting as a carrier for *ex vivo *expansion of corneal epithelial cells, and treatment of scleral melts/perforations.^65^ Many features of AMT make it an attractive adjunct in glaucoma surgery. Amniotic membrane transplantation can be utilized to repair leaking blebs following trabeculec-tomy surgery, to cover the plates of GDDs during initial placement, and to cover eroded GDD tube. Amniotic membrane can assist in decreasing postoperative fibrosis, leakage and vascularization around the trabeculectomy bleb, or the GDD plate.^68^

A 2015 Cochrane Review assessed 18 studies published up to December 2014 that studied outcomes of trabeculectomy with AMT. The review labeled the aggregation of data as low quality, but concluded that AMT may slightly improve IOP reduction and lead to slightly less frequent complications compared with standard trabeculectomy.^30^ Sheha et al,^69^ in a randomized prospective study of 37 eyes, compared trabeculectomy with MMC (control) to trabeculectomy with MMC and AMT (study group) and found that AMT had higher success rates, lower postoperative mean IOPs, and fewer complications. Khairy and Elsawy^70^ presented another prospective randomized controlled comparison trial of 52 eyes with trabeculectomy with MMC or with AMT. Results indicated that AMT was an acceptable alternative to MMC: over 24 months of follow-up, it was safe and effective in lowering IOP and had a reduced rate of postoperative complications.

Yazdani et al^68^ conducted a more recent randomized trial that was the first to evaluate the use of AMT compared with MMC in GDD surgery. The three arms were conventional Ahmed glaucoma valve (AGV), AGV with MMC, and AGV with AMT. A 3 × 3 cm single-layer AM was wrapped around the AGV plate with the aim of decreasing postoperative vascularization and fibrosis. The researchers found adjunctive AMT to be safe, but found no statistical difference in IOP reduction or complication rates compared with MMC in the 68 eyes examined.

Amniotic membrane transplantation can be used for late-onset glaucoma filtering bleb leakage. Rauscher et al^71^ compared bleb excision with conjunctival advancement to bleb excision with AMT. Initially, AMT had greater early leakage, but 1-year results showed that AMT was a suitable alternative to simply conjunctival advancement. Sethi et al^72^ assessed the use of AMT on patients with leaking blebs after trabeculectomy with MMC. Regarding surgical technique, a double-folded AM was draped over the leaking bleb with conjunctival advancement and preservation of the cystic bleb. They observed that the double-folded AM reinforced thin-walled blebs and promoted epithelization when the bleb bound to the membrane. This method was effective in all 17 eyes in restoring bleb function and stabilizing IOP visual acuity.^71^ Other studies found similar results.^73,74^

Finally, AMT can be utilized to cover donor sclera in cases of GDI erosions. Amniotic membrane transplantation has been used successfully in cases of recurrent erosion or excessive scarring restricting mobilization of the conjunctiva.^75^ A novel “sandwich” approach using amni-otic membrane that are shaped larger than the existing epithelial defect has yielded good results (Fig. 4). The first layer of amniotic membrane is subconjunctival, applied over the scleral patch with the epithelial side up (acting as the graft). The remaining conjunctiva is secured and a second layer of extraconjunctival amniotic membrane is applied epithelial side down (acting as the patch).^76,77^

The unit cost of AMT as of January 2017 is from around $550 to $900 depending on the size, type, and manufacturer. Due to the relatively high cost of AMT, it is typically reserved in current practice for operations in which conjunctival autograft is not readily available or feasibly extracted. While results over the years have been mixed to its effectiveness, recent studies have shown promising results that indicate AMT to be a safe and simple adjunct tool with an array of applications in the glaucoma surgical space.

## CONCLUSION

The utilization of adjunct tools for trabeculectomy and GDD has significant potential to improve outcomes in the surgical management of glaucoma. Collagen matrix implants (Ologen™), fibrin adhesives, and AMT provide several advantages when used in trabeculectomy, including the reduction of fibrosis and scarring during wound healing, reducing postoperative inflammation, and decreasing postoperative bleb leakage and hypotony. Although recent clinical trials have shown relatively positive results, there remains the need for larger, long-term clinical trials to confirm the effectiveness and long-term advantages of these adjunct tools in glaucoma surgeries. Ongoing research and development of innovative surgical tools and techniques will continue to improve the surgical approach for glaucoma to modulate wound healing, reduce complications, and improve outcomes of trabeculectomy and GDDs for patients.

## References

[1] Ichhpujani P, Dada T, Bhartiya S (2015). Biodegradable collagen implants in trabeculectomy.. J Curr Glaucoma Pract.

[2] Watson PG, Barnett F (1975). Effectiveness of trabeculectomy in glaucoma. Am J Ophthalmol.

[3] Gedde SJ, Herndon LW, Brandt JD, Budenz DL, Feuer WJ, Schiffman JC (2012). Tube Versus Trabeculectomy Study Group. Postoperative complications in the Tube Versus Trabeculectomy (TVT) study during five years of follow-up. Am J Ophthalmol.

[4] Gedde SJ, Schiffman JC, Feuer WJ, Herndon LW, Brandt JD (2012). Budenz DL; Tube versus Trabeculectomy Study Group. Treatment outcomes in the Tube Versus Trabeculectomy (TVT) study after five years of follow-up. Am J Ophthalmol.

[5] Chen CW, Huang HT, Bair JS, Lee CC (1990). Trabeculectomy with simultaneous topical application of mitomycin-C in refractory glaucoma. J Ocul Pharmacol.

[6] Lee SJ, Woo JM, Min JK, Kee CW, Yim JH (2012). The analysis of the clinical findings and effects of biodegradable collagen matrix in trabeculectomy. J Korean Ophthalmol Soc.

[7] Cillino S, Casuccio A, Di Pace F, Cagini C, Ferraro LL, Cillino G (2016). Biodegradable collagen matrix implant versus mitomycin-C in trabeculectomy: five-year follow-up. BMC Ophthalmol.

[8] Yuan F Li, L Chen X, Yan X, Wang L (2015). Biodegradable 3D-Porous Collagen Matrix (Ologen) compared with mitomy-cin C for treatment of primary open-angle glaucoma: results at 5 years. J Ophthalmol.

[9] Palanca-Capistrano AM, Hall J, Cantor LB, Morgan L, Hoop J, WuDunn D (2009). Long-term outcomes of intraoperative 5-fluorouracil versus intraoperative mitomycin C in primary trabeculectomy surgery. Ophthalmology.

[10] Anand N, Arora S, Clowes M (2006). Mitomycin C augmented glaucoma surgery: evolution of filtering bleb avascularity transconjunctival oozing and leaks. Br J Ophthalmol.

[11] Anand N, Atherley C (2005). Deep sclerectomy augmented with mitomycin C. Eye (Lond).

[12] Cillino S, Di Pace F, Cillino G, Casuccio A (2011). Biodegradable collagen matrix implant vs mitomycin-C as an adjuvant in trabeculectomy: a 24-month randomized clinical trial. Eye (Lond).

[13] DeBry PW, Perkins TW, Heatley G, Kaufman P, Brumback LC (2002). Incidence of late-onset bleb-related complications following trabeculectomy with mitomycin. Arch Ophthalmol.

[14] Jampel HD, Solus JF, Tracey PA, Gilbert DL, Loyd TL, Jefferys JL, Quigley HA (2012). Outcomes and bleb-related complications of trabeculectomy. Ophthalmology.

[15] Christakis PG, Tsai JC, Kalenak JW, Zurakowski D, Cantor LB, Kammer JA, Ahmed II (2013). The Ahmed versus Baerveldt study: three-year treatment outcomes. Ophthalmology.

[16] Budenz DL, Barton K, Gedde SJ, Feuer WJ, Schiffman J, Costa VP, Godfrey DG, Buys YM (2015). Ahmed Baerveldt Comparison Study Group. Five-year treatment outcomes in the Ahmed Baerveldt comparison study. Ophthalmology.

[17] Budenz DL, Feuer WJ, Barton K, Schiffman J, Costa VP, Godfrey DG, Buys YM (2016). Ahmed Baerveldt Comparison Study Group. Postoperative complications in the Ahmed Baerveldt comparison study during five years of follow-up. Am J Ophthalmol.

[18] Alvarado JA, Hollander DA, Juster RP, Lee LC (2008). Ahmed valve implantation with adjunctive mitomycin C and 5-fluorouracil: long-term outcomes. Am J Ophthalmol.

[19] Al-Mobarak F, Khan AO (2009). Two-year survival of Ahmed valve implantation in the first 2 years of life with and without intraoperative mitomycin-C. Ophthalmology.

[20] Amoozgar B, Lin SC, Han Y, Kuo J (2016). A role for antimetabolites in glaucoma tube surgery: current evidence and future directions. Curr Opin Ophthalmol.

[21] Cui QN, Hsia YC, Lin SC, Stamper RL, Rose-Nussbaumer J, Mehta N, Porco TC, Naseri A, Han Y (2017). Effect of mitomycin c and 5-flurouracil adjuvant therapy on the outcomes of Ahmed glaucoma valve implantation. Clin Experiment Ophthalmol.

[22] Dietlein TS, Lappas A, Rosentreter A (2013). Secondary subcon-junctival implantation of a biodegradable collagen-glycos-aminoglycan matrix to treat ocular hypotony following trabeculectomy with mitomycin C. Br J Ophthalmol.

[23] El-Saied HM, Abdelhakim MA (2016). Trabeculectomy with ologen in secondary glaucomas following failed trab-eculectomy with MMC: comparative study. Eye (Lond).

[24] Narayanaswamy A, Perera SA, Htoon HM, Hoh ST, Seah SK, Wong TT, Aung T (2013). Efficacy and safety of collagen matrix implants in phacotrabeculectomy and comparison with mitomycin C augmented phacotrabeculectomy at 1 year. Clin Exp Ophthalmol.

[25] Marey HM, Mandour SS, Ellakwa AF (2013). Subscleral trabecu-lectomy with mitomycin-C versus ologen for treatment of glaucoma. J Ocul Pharmacol Ther.

[26] Papaconstantinou D, Georgalas I, Karmiris E, Diagourtas A, Koutsandrea C, Ladas I, Apostolopoulos M, Georgopoulos G (2010). Trabeculectomy with OloGen versus trabeculectomy for the treatment of glaucoma: a pilot study. Acta Ophthalmol.

[27] Rosentreter A, Gaki S, Cursiefen C, Dietlein TS (2014). Trabeculectomy using mitomycin C versus an atelocollagen implant: clinical results of a randomized trial and histopathologic findings. Ophthalmologica.

[28] Rosentreter A, Schild AM, Jordan JF, Krieglstein GK, Dietlein TS (2010). A prospective randomised trial of trabeculec-tomy using mitomycin C vs an ologen implant in open angle glaucoma. Eye (Lond).

[29] Senthil S, Rao HL, Babu JG, Mandal AK, Garudadri CS (2013). Comparison of outcomes of trabeculectomy with mitomy-cin C vs. ologen implant in primary glaucoma. Indian J Ophthalmol.

[30] Wang X, Khan R, Coleman A (2015). Device-modified trabecu-lectomy for glaucoma. Cochrane Database Syst Rev.

[31] Perez CI, Mellado F, Jones A, Colvin R (2017). Trabeculectomy combined with collagen matrix implant (Ologen). J Glaucoma.

[32] Angmo D, Wadhwani M, Upadhyay AD, Temkar S, Dada T (2017). Outcomes of trabeculectomy augmented with subconjunctival and subscleral ologen implantation in primary advanced glaucoma. J Glaucoma.

[33] Chen HS, Ritch R, Krupin T, Hsu WC (2006). Control of filtering bleb structure through tissue bioengineering: an animal model. Invest Ophthalmol Vis Sci.

[34] Hsu WC, Ritch R, Krupin T, Chen HS (2008). Tissue bioengineering for surgical bleb defects: an animal study. Graefe’s Arch Clin Exp Ophthalmol.

[35] Hsu WC, Spilker MH, Yannas IV, Rubin PA (2000). Inhibition of conjunctival scarring and contraction by a porous collagen-glycosaminoglycan implant. Invest Ophthalmol Vis Sci.

[36] Rosentreter A, Mellein AC, Konen WW, Dietlein TS (2010). Capsule excision and Ologen implantation for revision after glaucoma drainage device surgery. Graefes Arch Clin Exp Ophthalmol.

[37] Martinowitz U, Spotnitz WD (1997). Fibrin tissue adhesives. Thromb Haemost.

[38] Koranyi G, Seregard S, Kopp ED (2004). Cut and paste: a no suture small incision approach to pterygium surgery. Br J Ophthalmol.

[39] Guo S, Wagner RS, Forbes BJ, DeRespinis PA, Caputo AR (2010). Cut and paste: sutureless conjunctival closure in strabismus surgery. J Pediatr Ophthalmol Strabismus.

[40] Batman C, Ozdamar Y, Aslan O, Sonmez K, Mutevelli S, Zilelioglu G (2008). Tissue glue in sutureless vitreoretinal surgery for the treatment of wound leakage. Ophthalmic Surg Lasers Imaging.

[41] O’ Sullivan F, Dalton R, Rostron CK (1996). Fibrin glue: an alternative method of wound closure in glaucoma surgery. J Glaucoma.

[42] Asrani SG, Wilensky JT (1996). Management of bleb leaks after glaucoma filtering surgery. Use of autologous fibrin tissue glue as an alternative. Ophthalmology.

[43] Sakarya Y, Sakarya R, Kara S, Soylu T (2011). Fibrin glue coating of the surgical surfaces may facilitate formation of a successful bleb in trabeculectomy surgery. Med Hypotheses.

[44] Panda A, Kumar S, Kumar A, Bansal R, Bhartiya S (2009). Fibrin glue in ophthalmology. Indian J Ophthalmol.

[45] Sharma A, Kaur R, Kumar S, Gupta P, Pandav S, Patnaik B, Gupta A (2003). Fibrin glue versus N-butyl-2-cyanoacrylate in corneal perforations. Ophthalmology.

[46] Almeida Junior GC, Arakawa L, Santi Neto DD, Cury PM, Lima Filho AA, Sousa SJ, Alves MR, Azoubel R (2015). Preoperative tranilast as adjunctive therapy to primary pterygium surgery with a 1-year follow-up. Arq Bras Oftalmol.

[47] Bahar I, Lusky M, Gaton D, Robinson A, Avisar R, Weinberger D (2006). The use of fibrin adhesive in trabeculectomy: a pilot study. Br J Ophthalmol.

[48] Bahar I, Weinberger D, Lusky M, Avisar R, Robinson A, Gaton D (2006). Fibrin glue as a suture substitute: histological evaluation of trabeculectomy in rabbit eyes. Curr Eye Res.

[49] Buschmann W, Stemberger A, Blumel G, Leydhecker W (1984). Fibrin adhesion and postoperative anti-fibrinolytic care of conjunctival wounds. Klin Monatsbl Augenheilkd.

[50] Choudhari NS, Neog A, Latka S, Srinivasan B (2015). Fibrin sealant-assisted revision of the exposed Ahmed tube. Middle East Afr J Ophthalmol.

[51] Choudhari NS, Neog A, Sharma A, Iyer GK, Srinivasan B (2013). Authors’ reply. Indian J Ophthalmol.

[52] Choudhari NS, Neog A, Sharma A, Iyer GK, Srinivasan B (2013). Our experience of fibrin sealant-assisted implantation of Ahmed glaucoma valve. Indian J Ophthalmol.

[53] Dal Pizzol MM, Roggia MF, Kwitko S, Marinho DR, Rymer S (2009). Use of fibrin glue in ocular surgery. Arq Bras Oftalmol.

[54] Dintelmann T, Lieb WE, Grehn F (2002). Filtering bleb revision. Techniques and outcome. Ophthalmologe.

[55] Du TT, Saffra N (2009). Acellular dermal graft as a treatment of recurrent conjunctival wound dehiscence. Arch Ophthalmol.

[56] Elmalem VI, Harris GJ (2012). Occurrence and surgical management of a cerebrospinal fluid-filled cystoid space following routine enucleation. Ophthal Plast Reconstr Surg.

[57] Esquenazi S, Rand W, Velazquez G, Grunstein L (2008). Novel therapeutic approach in the management of band keratopa-thy using amniotic membrane transplantation with fibrin glue. Ophthalmic Surg Lasers Imaging.

[58] Freeman PD, Kahook MY, Curtis TH (2010). Glaucoma drainage device implantation in children using fibrin glue. J AAPOS.

[59] Piltz JR, Starita RJ (1994). The use of subconjunctivally administered tissue plasminogen activator after trabeculectomy. Ophthalmic Surg.

[60] Por YM, Tan YL, Mehta JS, Tan DT (2009). Intracameral fibrin tissue sealant as an adjunct in tectonic lamellar keratoplasty for large corneal perforations. Cornea.

[61] Sakarya Y, Sakarya R, Yildirim A (2010). Sutureless amniotic membrane fixation with fibrin glue in symptomatic bullous keratopathy with poor visual potential. Eur J Ophthalmol.

[62] Lee GA, Holcombe DJ (2010). Surgical revision of dysfunctional filtration blebs with bleb preservation sliding conjunctival flap and fibrin glue. Eye (Lond).

[63] Kahook MY, Noecker RJ (2006). Fibrin glue-assisted glaucoma drainage device surgery. Br J Ophthalmol.

[64] Valimaki J (2006). Fibrin glue for preventing immediate postoperative hypotony following glaucoma drainage implant surgery. Acta Ophthalmol Scand.

[65] Malhotra C, Jain AK (2014). Human amniotic membrane transplantation: different modalities of its use in ophthalmology. World J Transplant.

[66] Dua HS, Gomes JA, King AJ, Maharajan VS (2004). The amni-otic membrane in ophthalmology. Surv Ophthalmol.

[67] Shimmura S, Shimazaki J, Ohashi Y, Tsubota K (2001). Anti-inflammatory effects of amniotic membrane transplantation in ocular surface disorders. Cornea.

[68] Yazdani S, Mahboobipour H, Pakravan M, Doozandeh A, Ghahari E (2016). Adjunctive mitomycin c or amniotic membrane transplantation for Ahmed glaucoma valve implantation: a randomized clinical trial. J Glaucoma.

[69] Sheha H, Kheirkhah A, Taha H (2008). Amniotic membrane transplantation in trabeculectomy with mitomycin C for refractory glaucoma. J Glaucoma.

[70] Khairy HA, Elsawy MF (2015). Trabeculectomy with mitomycin-C versus trabeculectomy with amniotic membrane transplant: a medium-term randomized controlled trial. J Glaucoma.

[71] Rauscher FM, Barton K, Budenz DL, Feuer WJ, Tseng SC (2007). Long-term outcomes of amniotic membrane transplantation for repair of leaking glaucoma filtering blebs. Am J Ophthalmol.

[72] Sethi P, Patel RN, Goldhardt R, Ayyala RS (2016). Conjunctival advancement with subconjunctival amniotic membrane draping technique for leaking cystic blebs. J Glaucoma.

[73] Kitagawa K, Yanagisawa S, Watanabe K, Yunoki T, Hayashi A, Okabe M, Nikaido T (2009). A hyperdry amniotic membrane patch using a tissue adhesive for corneal perforations and bleb leaks. Am J Ophthalmol.

[74] Nagai-Kusuhara A, Nakamura M, Fujioka M, Negi A (2008). Long-term results of amniotic membrane transplantation-assisted bleb revision for leaking blebs. Graefes Arch Clin Exp Ophthalmol.

[75] Rai P, Lauande-Pimentel R, Barton K (2005). Amniotic membrane as an adjunct to donor sclera in the repair of exposed glaucoma drainage devices. Am J Ophthalmol.

[76] Ainsworth G, Rotchford A, Dua HS, King AJ (2006). A novel use of amniotic membrane in the management of tube exposure following glaucoma tube shunt surgery. Br J Ophthalmol.

[77] Papadaki TG, Siganos CS, Zacharopoulos IP, Panteleontidis V, Charissis SK (2007). Human amniotic membrane transplantation for tube exposure after glaucoma drainage device implantation. J Glaucoma.

